# Combined effect of methanolic extracts of *Senna occidentalis* (Fabaceae) and *Khaya senegalensis* (Meliaceae) collected in the far-north region (Cameroon) on adults of *Anopheles gambiae* senso stricto GILES 1902

**DOI:** 10.1016/j.parepi.2025.e00450

**Published:** 2025-07-17

**Authors:** Ngatarang Celestine, Nanga Woulsou Maurice, Saotoing Pierre, Ndjonka Dieudonné

**Affiliations:** a Department of Life and Earth Sciences, Teachers' Training College, University of Maroua, Cameroon; b Department of Biological Sciences, Faculty of Science, University of Ngaoundéré, Cameroon

**Keywords:** Adulticide, Phytoextracts, *Anopheles gambiae* ss, *Senna occidentalis*, *Khaya senegalensis*

## Abstract

**Background and objectives:**

Malaria remains a significant public health burden in sub-Saharan Africa, primarily due to the presence of its primary vector, *Anopheles gambiae*. The increasing resistance to synthetic insecticides has necessitated the exploration of environmentally friendly alternatives, such as plant-derived bioactive compounds. This study investigated the adulticidal activity of methanolic extracts from the leaves of *Senna occidentalis* (Fabaceae) and *Khaya senegalensis* (Meliaceae) against *A. gambiae*, a primary malaria vector, in a laboratory experiment conducted from January to February 2020 at the Entomology Laboratory, University of Ngaoundéré, Adamaoua Region, Cameroon.

**Methodology and results:**

The extraction yields were 17.8 % for *S. occidentalis* and 14.9 % for *K. senegalensis*. Phytochemical analysis revealed that *S. occidentalis* leaves contained 21.55 mg gallic acid equivalent (GAE)/100 g of polyphenols and 24.88 mg quercetin equivalent (QE)/100 g of flavonoids, whereas *K. senegalensis* leaves contained 18.34 mg GAE/100 g of polyphenols, 34.34 mg QE/100 g of flavonoids, and 27.56 mg catechin equivalent (CE)/100 g of tannins. Bioassays conducted according to World Health Organization (WHO) protocols demonstrated synergistic insecticidal effects between the extracts against *A. gambiae*. The lethal concentration (LC_50_) values were 0.87 g/L for *S. occidentalis* and 1.07 g/L for *K. senegalensis*, with half-lethal time (HL_50_) values of 2 h 48 min and 2 h 14 min, respectively. The most effective combination (*S. occidentalis* [75 %] + *K. senegalensis* [25 %]) achieved an LC_50_ of 0.98 g/L and HL_50_ of 1 h 45 min.

**Conclusion and application:**

These results highlight the potential of *S. occidentalis* and *K. senegalensis* extracts as sustainable biocides for controlling *A. gambiae* ss populations. Their synergistic efficacy and high phytochemical content support further development into eco-friendly vector control tools, aligning with global efforts to combat insecticide resistance.

## Highlights

• Combinations of *Senna occidentalis* and *Khaya senegalensis* leaf extracts exhibit adulticidal effects against *Anopheles gambiae* ss.
• P1 75 % + P2 25 % combination is the most effective, with LC_50_ and HL_50_ values of 0.98 g/L and 1 h 45 min, respectively.
• Concentration- and time-dependent mortality rate inidcate potential for natural biocide development.
• Phytochemical analysis confirms presence of insecticidal secondary metabolites.
• Natural extracts offer promising alternative for malaria vector control.

## 1 Introduction

Malaria, a febrile disease caused by Plasmodium parasites, is transmitted to humans through the bite of an infected female Anopheles mosquito (Amang à Ngnoung et al., 2023; Ayo et al., 2021; Duffy et al., 2024). Despite efforts to control it, malaria remains a significant public health concern, particularly in sub-Saharan Africa, where it is responsible for an estimated 216 million cases annually, with 81 % of these cases occurring in sub-Saharan Africa (Liu et al., 2014; Loy et al., 2017; Snow et al., 2017). Children under the age of 5 and pregnant women are the most vulnerable populations. In Cameroon, malaria is a major endemic disease, accounting for 40–50 % of medical consultations, 23 % of hospital admissions, and approximately 40 % of the annual household health budget (Nguiffo-Nguete et al., 2023).

Several challenges hinder malaria control efforts, including the emergence of Plasmodium resistance to antimalarial drugs, mosquito resistance to insecticides, poor hygiene conditions, economic constraints, and inadequate implementation of preventive and curative measures (Amang à Ngnoung et al., 2023; Ayo et al., 2021; Espinoza, 2025; Nganso Ditchou et al., 2020; Nguiffo-Nguete et al., 2023). In response to these challenges, the World Health Organization advocates for new strategies to combat malaria. One effective approach is vector population control, specifically targeting the Anopheles mosquito species responsible for disease transmission (Aviña-Zubieta et al., 2025; Kacholi, 2024; WHO, 2015). Current vector control methods, such as impregnated mosquito nets and indoor spraying with synthetic insecticides, have limitations due to their potential harm to humans, animals, and the environment (Abubakar et al., 2021; Duffy et al., 2024; Hamadou et al., 2022; Nguiffo-Nguete et al., 2023). Therefore, there is an urgent need to identify natural, biodegradable, and locally available insecticides.

**Fig. 1:**
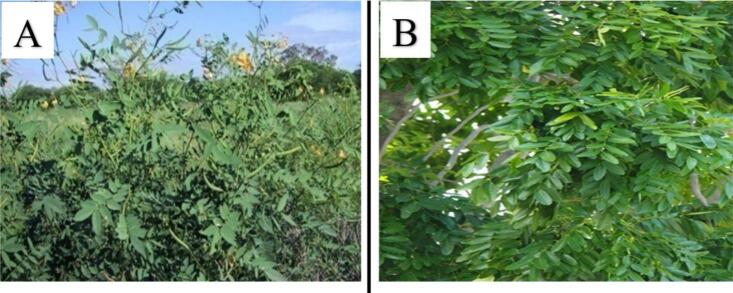
*Senna occidentalis* Linn (A) and *Khaya senegalensis* (B)

Botanical insecticides offer a promising alternative due to their biodegradability, low non-target toxicity, and cultural acceptability (Amang à Ngnoung et al., 2023; Mogaka et al., 2023b; Oscar Ditchou Nganso et al., 2020b; WHO, 2005). In Cameroon, plants like *Senna occidentalis* (Fabaceae) and *Khaya senegalensis* (Meliaceae) have long been utilized in traditional practices for their insect-repellent properties. *Senna occidentalis* L., a suffrutescent pantropical species native to South America, thrives in disturbed habitats and is characterized by paripinnate leaves, yellow inflorescences, and pods containing toxic seeds (Fig. 1A). Ethnobotanical studies highlight its use in repelling mosquitoes and treating parasitic infections (Lum Nde et al., 2022; Mogaka et al., 2023a). Similarly, *Khaya senegalensis* (Desv.) A.Juss., a towering deciduous tree native to the Sudanian savanna, features scaly bark, compound leaves, and winged seeds (Fig. 1B). Its bark and leaf extracts are traditionally employed to deter insects and manage fevers (Amang à Ngnoung et al., 2023; Oscar Ditchou Nganso et al., 2020a).

The use of natural substances with proven insecticidal properties is increasingly encouraged. In African traditional medicine, certain plants have been used for centuries to control insect vectors (Hamadou et al., 2020; Mamoudou et al., 2024a; Mamoudou and Mune Mune, 2024; Nganso Ditchou et al., 2020; WHO, 2016). The present study aims to contribute to malaria eradication by reducing the Anopheles population. We selected *Senna occidentalis* and *Khaya senegalensis* for their potential insecticidal and repellent effects, as they are traditionally used by local populations to deter mosquitoes and flies.

## 2 Materials and methods

### 2.1 Methodology

#### 2.1.1 Plant material

This study employed two plant species, namely *Senna occidentalis* (Fig. 1A) and *Khaya senegalensis* (Fig. 1B). *S. occidentalis* Linn is an herbaceous plant characterized by its erect growth habit, reaching heights of 0.5–1.5 m, with glabrescent and paripinnate leaves measuring 15–20 cm in length, and emitting a distinct fragrance. In contrast, *K. senegalensis*, commonly referred to as African mahogany or Senegal mahogany, is a tree species belonging to the Meliaceae family, distinguished by its unique morphological characteristics.

#### 2.1.2 Harvesting of plant material

The plant material was collected in September 2019 between 6:00 and 9:00 a.m. in the Pitoaré and Djarengol districts, specifically at Camp Agricole in Maroua, Far North, Cameroon. The collected specimens were identified at the Yaoundé National Herbarium and assigned the following herbarium numbers: *Senna occidentalis* (42,060/HNC) and *Khaya senegalensis* (18,628/SRF/Cam). After collection, the leaves were shade-dried for 10 days to reduce moisture content. The dried leaves were then ground into fine powders using a mortar.

#### 2.1.3 Extraction of active ingredients

**Fig. 2:**
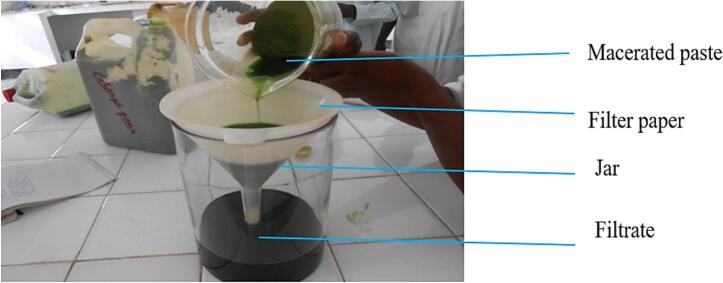
Extraction of active ingredients.

A pure methanolic maceration extraction method was employed to isolate the active ingredients from the plant materials (Avanza et al., 2021; Hamadou et al., 2020; Mamoudou et al., 2024b). This process was carried out at the Chemistry Laboratory of the Teachers' Training College of Maroua. Methanol was selected as the solvent due to its high solubility, ability to transfer materials, and capacity to penetrate plant cells. The required quantities of leaf powder were accurately weighed using a HCB123 electronic balance with a precision of ±0.001 g. Specifically, 500 g of powder from each plant was weighed and macerated in 2 l of methanol for 24 h (Amang à Ngnoung et al., 2023; Bayang et al., 2025; Wangso et al., 2022). The resulting mixture was homogenized through manual agitation to facilitate the release of active molecules from the plant material. The mixture was then filtered using filter paper inserted into a funnel (Fig. 2).

The filtrates were then concentrated using a rotavapor to separate the solvent from the active molecules released from the leaves and bark. This process involved placing the filtrate in a water-bath flask connected to the rotavapor (Amang à Ngnoung et al., 2023; Wangso et al., 2022). The water bath was set to a temperature range of 78–80 °C, close to the boiling point of methanol. Upon activation of the rotavapor, the solvent was evaporated and released in vapor form (Amang à Ngnoung et al., 2023; Wangso et al., 2022). The vapor was then condensed and collected in a separate flask as pure methanol. Meanwhile, the water bath flask retained only the active ingredient required for insecticide testing.

The yield (R) of the extraction process was calculated using the following formula:

$$R\left(\%\right)=\frac{\text{mass of active ingredient}\;}{\text{total mass of powder}}\times 100$$

#### 2.1.4 Breeding *Anopheles gambiae* sensu stricto

**Fig. 3:**
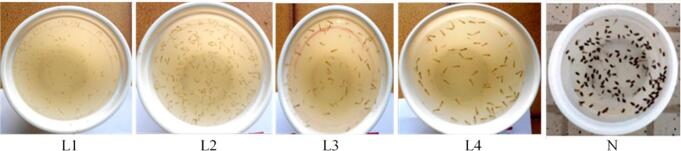
Different larval stages resulting from the hatching of *Anopheles gambiae* ss eggs. **Legend.** L1: stage I larvae; L2: stage II larvae; L3: stage III larvae; L4: stage IV larvae; N: pupae

**Fig. 4:**
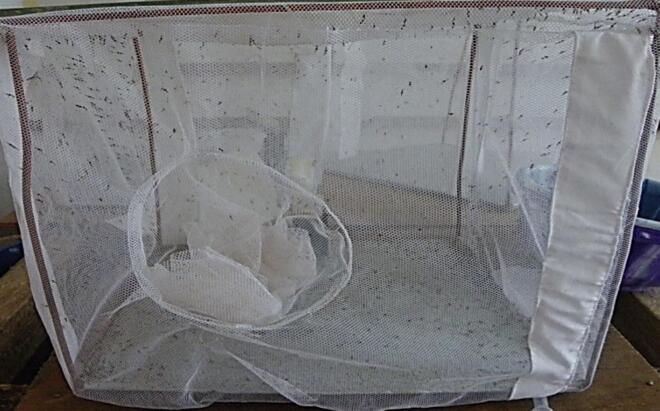
Newly emerged Anopheles adults

A laboratory strain of *A. gambiae* ss was obtained from the “*Organisation pour la Coordination des Endémies en Afrique Central”* (OCEAC, Yaoundé, Cameroon). The eggs were soaked in borehole water to facilitate hatching (WHO, 2016; WHO, 2013; WHO, 2005). After 5 h, a few eggs hatched, yielding stage I (LI) larvae, approximately 2 mm in length. Over the next 2 days, the stage I larvae developed into stage II larvae, ranging in size from 2 to 3 mm (Fig. 3). The larvae continued to progress through various stages, ultimately reaching stage IV larvae within 7–8 days after egg soaking (Fig. 4). Stage IV larvae underwent metamorphosis after 3 days, resulting in pupae. Different larval stages were separated and transferred to plastic cups (Fig. 4) for rearing. The larvae were fed a nutrient-rich diet supplied by OCEAC, consisting of TetraMin® (Tetra GmbH, Germany), a mixture of shrimp powder, mollusc, and vitamins A and B. The nutrient solution was sprayed onto the water containing the various aquatic stages, and the water was renewed every 2 days to prevent pollution. The larval density was maintained at 100 larvae per 100 mL of water.

The insectarium was maintained at a constant temperature of 28.2 °C ± 0.9 °C and a relative humidity of 80 % using a radiator. Pupae were collected using a bulb pipette and transferred to plastic cups containing rearing water, then introduced into rearing cages designed according to the World Health Organization (WHO) model, measuring 30 cm × 30 cm × 30 cm (WHO, 2016; WHO, 2013; WHO, 2005).

#### 2.1.5 Preparation of test solutions

The concentrations used in this study were selected based on a preliminary test and followed the WHO method for assessing the sensitivity of mosquito larvae and adults to insecticides (WHO, 2016, WHO, 2013, WHO, 2005). A 39 g/L stock solution was prepared, which was then diluted to create four test concentrations: 500 ppm, 1500 ppm, 2500 ppm, 3500 ppm, and 5000 ppm. Two control concentrations were also prepared: a zero control (farm water) and a positive control (Deltamethrin at 55 ppm), which served as the standard for adulticide tests.

#### 2.1.6 Adulticide test

The adulticidal activity of the test solutions was assessed according to standard CDC and WHO procedures (Aïzoun et al., 2013; Peard et al., 2024; WHO, 2005, WHO, 2013, WHO, 2016) using 250 mL test bottles. The bottles were thoroughly washed and dried, then labeled with the corresponding concentration numbers. For each concentration, a test volume was taken from the stock solution using a pipette and introduced into the bottle. The bottles were then tightly closed and turned to homogenize the interior. After homogenization, the bottles were placed in vertical positions to dry the interior.

**Fig. 5:**
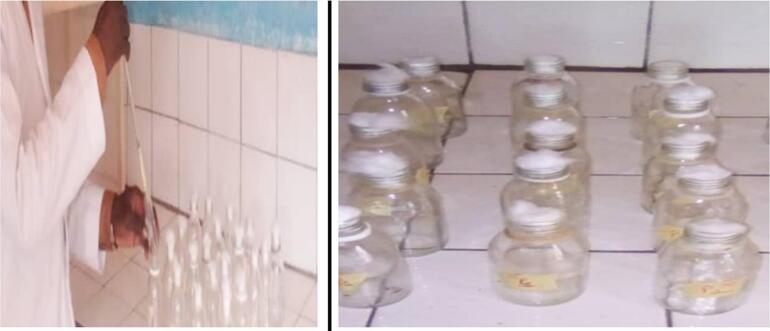
Adulticide test device.

Twenty (20) unsexed 4-day-old *A. gambiae* ss adults were introduced into each bottle using a mouth aspirator. Once the first mosquitoes were introduced into the bottle, a stopwatch was started, and observations began to count the number of dead mosquitoes. Observations were made every 30 min until the 6th hour, then every 1 h until the 12th hour of exposure. Finally, the device was abandoned to carry out the last observation after 24 h of exposure (Fig. 5). A mosquito was considered dead when, after turning the bottle carefully for 3 to 4 min, it was visibly no longer moving.

The number of deaths was transformed into a mortality rate, then classified into hourly bands of 1 h, 6 h, 12 h, 18 h, and 24 h of exposure. For each concentration, the test was repeated three times, and the mortality rates were used to plot curves showing the evolution of mosquito mortality.

Combined testing of crude extracts was carried out using the percentage combination model, according to the CDC protocol revised by WHO (Aïzoun et al., 2013; Peard et al., 2024; WHO, 2013, WHO, 2016).

**Plant 1** (75 %) + Plant 2 (25 %).

**Plant 1** (25 %) + Plant 2 (75 %).

**Plant 1** (50 %) + Plant 2 (50 %).

**Plant 1:** *Senna occidentalis.*

**Plant 2:** *Khaya senegalensis*

#### 2.1.7 Data analysis

GraphPad software was used to generate curves illustrating the changes in mortality as a function of concentration and time. Histograms and regression lines were produced using Microsoft Excel 2013 to determine the lethal concentration 50 (LC_50_) and half-lethal time (HL_50_) values (Aïzoun et al., 2013; Hoekstra, 1987). The LC_50_ (Eq. 1) and HL_50_ (Eq. 2) values for the plant extracts were calculated using the equation of Finney (Finney, 1952).

(1) $${\mathrm{CL}}_{50}=\log\;10^{-1}\;\left(\frac{5-b}{a}\right)$$

(2) $${\mathrm{HL}}_{50}=\log\;10^{-1}\left(\frac{5-b}{a}\right);y=\mathrm{ax}+b$$

## 3 Results

### 3.1 Extraction yield

The results presented in Table 1 reveal the extraction data of two plant species, *S. occidentalis* and *K. senegalensis*. Notably, the extraction yield of *S. occidentalis* leaves, harvested on September 2nd, 2019, was significantly higher (17.8 %) compared to *K. senegalensis* leaves, harvested on September 20th, 2019 (14.6 %). This discrepancy in extraction efficiency may be attributed to variations in plant maturity, environmental conditions, or inherent species-specific characteristics. Furthermore, the mass of extract obtained from *S. occidentalis* (89 g) was greater than that of *K. senegalensis* (73 g), despite both species having the same initial mass of 500 g. These findings suggest that *S. occidentalis* may be a more promising species for extract production, and further investigation into the optimal harvesting conditions and extraction protocols for both species is warranted to maximize yields and efficiencies.

**Table 1:** Extraction yield.

Species	Organs	Mass powder (g)	Mass of extract (g)	Rd (%)
*Senna occidentalis*	Leaves	500	89	17.80
*Khaya senegalensis*	Leaves	500	73	14.60

### 3.2 Phytochemical analysis of plant species

The qualitative phytochemical test results presented in Table 2 reveal the presence and relative abundance of various phytochemical compounds in the extracts of *S. occidentalis* and *K. senegalensis*. Notably, both species exhibited a moderate to high presence of polyphenols, terpenoids, and flavonoids, indicating a rich chemical diversity. However, differences in the relative abundance of these compounds were observed between the two species. *S. occidentalis* exhibited a higher presence of tannins (+++) and a moderate presence of saponins (++), whereas *K. senegalensis* showed a higher presence of flavonoids (+++) and a lower presence of saponins (+). These findings suggest that the two species may have distinct phytochemical profiles, which could be related to their specific biological activities and potential therapeutic applications.

**Table 2:** Qualitative phytochemical test results.

Species	Polyphenols	Terpenoids	Flavonoids	Saponins	Tannins
*Senna occidentalis*	+++	++	++	++	+++
*Khaya senegalensis*	++	++	+++	+	++

The quantitative phytochemical test results presented in Table 3 provide a detailed analysis of the phytochemical composition of *S. occidentalis* (SO) and *K. senegalensis* (KS) extracts.

**Table 3:** Quantitative phytochemical test results.

Plants	Polyphenols (g GAE/100 g DM)	Flavonoids (g QE/100 g DM)	Tannins (g CE/100 g DM)	Saponins (g GAE/100 g DM)	Terpenoids (g LE/100 g DM)
KS	18.34 ± 0.84	3.43 ± 0. 32	2.75 ± 0.20	3.30 ± 0.69	10.63 ± 0.56
SO	21.55 ± 0.47	2.48 ± 0.07	6.55 ± 0.40	4.60 ± 0.64	14.24 ± 0.52

KS: Khaya senegalensis; SO: Senna occidentalis.

The results show that both plants are rich in polyphenols, with SO exhibiting a significantly higher content (21.55 ± 0.47 g GAE/100 g DM) compared to KS (18.34 ± 0.84 g GAE/100 g DM) (Table 3). Conversely, KS had a higher flavonoid content (3.43 ± 0.32 g QE/100 g DM) compared to SO (2.48 ± 0.07 g QE/100 g DM). The tannin content was significantly higher in SO (6.55 ± 0.40 g CE/100 g DM) compared to KS (2.75 ± 0.20 g CE/100 g DM), while the saponin content was also higher in SO (4.60 ± 0.64 g GAE/100 g DM) compared to KS (3.30 ± 0.69 g GAE/100 g DM). Finally, the terpenoid content was higher in SO (14.24 ± 0.52 g LE/100 g DM) compared to KS (10.63 ± 0.56 g LE/100 g DM).

These findings suggest that SO may have a more pronounced antioxidant and anti-inflammatory potential due to its higher polyphenol and tannin content, while KS may have a more significant antimicrobial activity due to its higher flavonoid content.

### 3.3 Adulticidal effect of the P1 25 % + P2 75 % combination on *Anopheles gambiae* ss

**Fig. 6:**
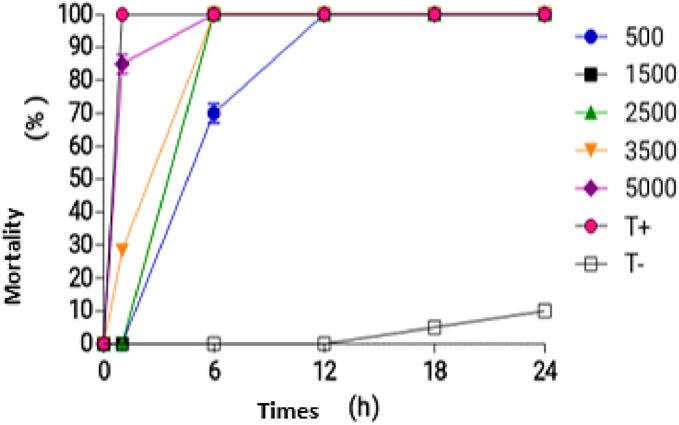
Temporal evolution of mortality rates in *Anopheles gambiae* sensu stricto adults exposed to a combined treatment of 25 % *Senna occidentalis* (SO) and 75 % *Khaya senegalensis* (KS) methanolic extracts.

Fig. 6 illustrates the time-course of adult *A. gambiae* mortality rates in response to various concentrations of the combined extracts from the two plants. Following 1 h of exposure, the different concentrations (500 ppm, 1500 ppm, 2500 ppm, 3500 ppm, and 5000 ppm) resulted in 0 %, 0 %, 30 %, 60 %, and 80 % mortality of Anopheles adults, respectively, whereas the positive control achieved 100 % mortality. Notably, all concentrations induced 100 % adult mortality after 12 h of exposure.

**Fig. 7:**
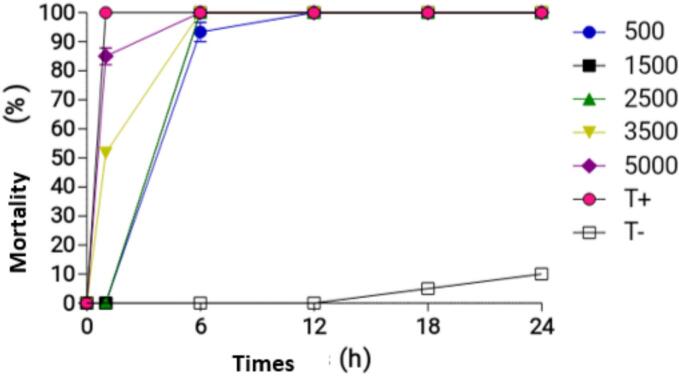
Mortality rates of adult Anopheles exposed to the combination of P1 50 % + P2 50 %.

For the P1 50 % + P2 50 % combination, the different concentrations induced the following mortality rates after 1 h of exposure: 0 %, 0 %, 50 %, and 85 %, respectively. After 6 h of exposure, the mortality rates increased to 90.00 % (500 ppm), 93.33 % (1500 ppm), 96.66 %, and 100.00 % (3500 ppm and 5000 ppm), respectively. Notably, all concentrations achieved 100 % mortality after 12 h of exposure (Fig. 7).

**Fig. 8:**
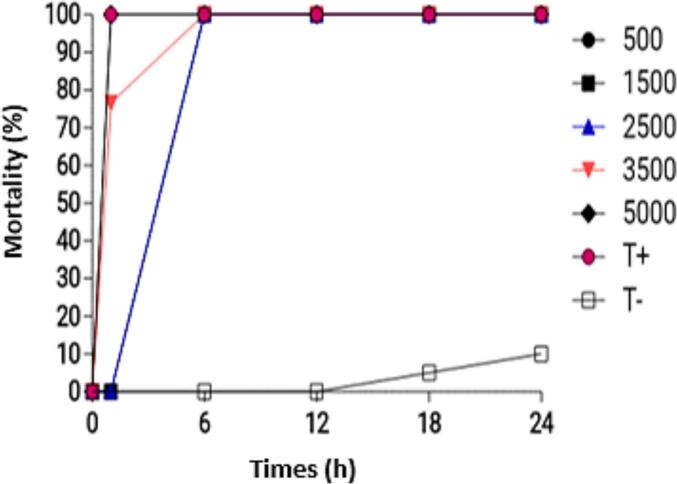
Evolution of adult Anopheles mortality rates with the combination of P1 75 % + P2 25 %.

The evolution of mortality rates of anophelines exposed to the combination of P1 75 % + P2 25 % (Fig. 8) shows that all concentrations induced 100 % mortality after 1 h of exposure.

The results presented in Table 4 demonstrate the synergistic effects of combining methanolic extracts of *K. senegalensis* (P1) and *S. occidentalis* (P2) on the lethal concentration (LC_50_) and hematolytic activity (HL_50_). The data reveals that varying the proportions of P1 and P2 in the combination significantly impacts the toxicity and hemolytic activity of the resulting mixture.

**Table 4:** LC_50_ and HL_50_ of the combined effects of methanolic extracts of *K. senegalensis* and *S. occidentalis*.

Combinations	Equations	R	LC_50_ (g/L)	HL_50_
P1 75 % + P2 25 %	Y = x + 5.0045	0.66	0.98	1h45min
P1 25 % + P2 75 %	Y = x + 4.875	0.87	1.33	2h13min
P1 50 % + P2 50 %	Y = x + 4.865	0.85	1.36	2h45min

HL_50_: Half-lethal time (hours:minutes); LC_50_: Lethal concentration (g/L); P1: Proportion of *Senna occidentalis*; P2: Proportion of *Khaya senegalensis*.

The combination of 75 % P1 and 25 % P2 (P1 75 % + P2 25 %) exhibited the lowest LC_50_ value of 0.98 g/L, indicating the highest toxicity among the three combinations tested (Table 4). This suggests that the dominant presence of P1 in this combination contributes to the enhanced toxicity. Furthermore, this combination also displayed the shortest HL_50_ time of 1h45min, indicating the fastest hemolytic activity. The rapid hemolysis observed in this combination may be attributed to the synergistic interaction between the bioactive compounds present in P1 and P2.

In contrast, the combination of 25 % P1 and 75 % P2 (P1 25 % + P2 75 %) showed a significantly higher LC_50_ value of 1.33 g/L (Table 4), indicating a reduced toxicity compared to the previous combination. This suggests that the higher proportion of P2 in this combination may be responsible for the decreased toxicity. Additionally, this combination exhibited a longer HL_50_ time of 2h13min, indicating a slower hemolytic activity. The reduced hemolytic activity observed in this combination may be attributed to the antagonistic interaction between the bioactive compounds present in P1 and P2.

The combination of equal proportions of P1 and P2 (P1 50 % + P2 50 %) exhibited an intermediate LC_50_ value of 1.36 g/L and an HL_50_ time of 2h45min (Table 4). This suggests that the equal presence of P1 and P2 in this combination results in a balanced toxicity and hemolytic activity.

The correlation coefficients (R) for each combination were found to be 0.66, 0.87, and 0.85 for P1 75 % + P2 25 %, P1 25 % + P2 75 %, and P1 50 % + P2 50 %, respectively (Table 4). These values indicate a moderate to strong positive correlation between the concentration of the extracts and the resulting toxicity and hemolytic activity.

Overall, the results suggest that the combination of 75 % P1 and 25 % P2 exhibits the most potent toxic and hemolytic effects, while the combination of 25 % P1 and 75 % P2 has the least potent effects. The equal combination of P1 and P2 has intermediate effects.

## 4 Discussion

The results of the phytochemical analyses revealed that the same plant parts harvested from different locations can contain distinct secondary metabolites, even when identical extraction techniques are employed. This variation is attributed to several factors, including soil type, climate, harvesting period, and the plant's state during harvesting. This finding is consistent with the work of Kolawole et al. (2011)*,* Amang à Ngnoung et al. (2023), and Leutcha et al. (2025) who reported similar differences in the secondary metabolites of ethanolic extracts from the leaves of *Khaya senegalensis*, *Tithonia diversifolia, Cyperus rotundus*, and *Hyptis suaveloensis* in Nigeria. Similarly, Nganso Ditchou et al. (2020), Kolawole et al. (2011), and Nganso Ditchou et al. (2024) demonstrated that extracts from plants harvested in different regions of Mali, including *Cassia occidentalis*, contained distinct secondary metabolites.

The methanolic extracts of *S. occidentalis* and *K. senegalensis* were found to contain secondary metabolites such as terpenoids, tannins, and flavonoids, which are classified as pyrethrenoids. These extracts likely induce neurotoxic effects on adult Anopheles, as reported for related botanical compounds targeting insect acetylcholine esterase activity (Adedayo et al., 2022). This mode of action supports their potential as adulticides. This finding is supported by previous studies, including those by Lum Nde et al. (2022), Badock et al. (2024), Amang à Ngnoung et al. (2023), and Mogaka et al. (2023b) which demonstrated the adulticidal effects of plant leaf extracts on Anopheles and other mosquito species.

The comparative analysis of the insecticidal effects of *S. occidentalis* and *K. senegalensis* extracts on adults of *A. gambiae* ss revealed that *S. occidentalis* extracts exhibited a higher toxicity than those of *K. senegalensis* (Amang à Ngnoung et al., 2023; Mamoudou et al., 2025; Mogaka et al., 2023a, Mogaka et al., 2023b; Nandwa et al., 2024; Oscar Ditchou Nganso et al., 2020a). This disparity in toxicity may be attributed to the differential composition of chemical compounds responsible for insecticidal effects, as observed during the phytochemical analysis. Specifically, the extracts of *S. occidentalis* contained higher levels of terpenoids, flavonoids, tannins, and saponins, which are known to possess neurotoxic effects on Anopheles.

A limited number of studies have investigated the insecticidal effects of these two plants. However, the work of Lum Nde et al. (2022), Badock et al. (2024) demonstrated that *S. occidentalis* extracts were more effective than *Trichilia emecta* and *Erythrina senegalensis* extracts in controlling adults of *A. gambiae* ss. In a comparative assessment of the efficacy of the different combinations of extracts, the P1 75 % + P2 25 % combination was found to be the most effective against *A. gambiae* ss adults.

The synergistic effect of the secondary metabolites presents in the extracts of *S. occidentalis* and *K. senegalensis* is thought to be responsible for the observed adulticidal effect. The combined action of these metabolites, including terpenoids, flavonoids, tannins, and saponins, may have contributed to the enhanced toxicity of the extracts. This hypothesis is supported by the work of Oscar Ditchou Nganso et al. (2020a), Kolawole et al. (2011), Tang et al. (2024), Kacholi Kacholi (2024), and Aviña-Zubieta et al. (2025), which demonstrated the role of carboxylesterase in the insecticidal activity of certain plant extracts. In addition, synergistic effects were observed in the *S. occidentalis* (75 %) + *K. senegalensis* (25 %) combination, with an LC_50_ of 0.98 g/L—lower than the theoretical additive LC_50_ (1.02 g/L) calculated using independent action models (Finney, 1952; WHO, 2005). These findings indicate a synergistic effect that exceeds simple additive interactions, in line with the phytochemical complementarity observed in plant-derived insecticides, as documented by several studies (Raimi et al., 2024; Sarma et al., 2019; Wangrawa et al., 2023).

Furthermore, the results of the present study are consistent with the findings of Muturi et al. (2017) and Sarma et al. (2019), which demonstrated the additive effect of combining multiple essential oils on the larvicidal potential of *Aedes aegypti* larvae. Similarly, the work of Youssefi et al. (2019) showed that the combination of two monoterpenoids, carvacrol and thymol, resulted in a significant enhancement of their individual larvicidal effects against chaga disease vector larvae.

The high mortality rate observed in the present study may be attributed to the toxicity of the various metabolites present in the extracts of *S. occidentalis* and *K. senegalensis*. This hypothesis is supported by the work of Amang à Ngnoung et al. (2023), which demonstrated the insecticidal effects of plant extracts on mosquito larvae.

## 5 Conclusion

This study evaluated the adulticidal effect of various combinations of *Senna occidentalis* and *Khaya senegalensis* leaf extracts on the *A. gambiae* ss strain, using the CDC/WHO protocol and test bottles. The results demonstrated that the combinations exhibited significant adulticidal potential against *A. gambiae* ss. Notably, the P1 75 % + P2 25 % combination was the most effective, with LC_50_ and HL_50_ values of 0.98 g/L and 1 h 45 min, respectively. Mortality rates were found to be concentration- and time-dependent. Based on these findings, the combination of *S. occidentalis* and *K. senegalensis* extracts is strongly recommended for the development of natural biocides in vector control against malaria and other parasitic diseases.

## CRediT authorship contribution statement

**Ngatarang Celestine:** Writing – review & editing, Writing – original draft, Methodology, Investigation, Formal analysis, Conceptualization. **Nanga Woulsou Maurice:** Writing – review & editing, Investigation, Formal analysis. **Saotoing Pierre:** Writing – review & editing, Visualization, Validation, Supervision, Project administration, Methodology. **Ndjonka Dieudonné:** Writing – review & editing, Visualization, Validation, Supervision.

## Funding

No funding.

## Declaration of competing interest

No conflict of interest.

## References

[bb0005] Abubakar A.M., Soltanifar Z., Luka Y., Udoh E.W., Hamadou M. (2021). Analysis of microbial growth models for microorganisms in chicken manure digester. Int. J. Res. Sci. Eng..

[bb0010] Adedayo B.C., Ogunsuyi O.B., Akinniyi S.T., Oboh G. (2022). Effect of Andrographis paniculata and Phyllanthus amarus leaf extracts on selected biochemical indices in Drosophila melanogaster model of neurotoxicity. Drug Chem. Toxicol..

[bb0015] Aïzoun N., Ossè R., Azondekon R., Alia R., Oussou O., Gnanguenon V., Aikpon R., Padonou G.G., Akogbéto M. (2013). Comparison of the standard WHO susceptibility tests and the CDC bottle bioassay for the determination of insecticide susceptibility in malaria vectors and their correlation with biochemical and molecular biology assays in Benin, West Africa. Parasit. Vectors.

[bb0020] Amang à Ngnoung G.A., Nganso Ditchou Y.O., Leutcha P.B., Dize D., Tatsimo S.J.N., Tchokouaha L.R.Y., Kowa T.K., Tembeni B., Mamoudou H., Poka M., Demana P.H., Siwe Noundou X., Fekam Boyom F., Meli Lannang A. (2023). Antiplasmodial and antileishmanial activities of a new limonoid and other constituents from the stem bark of Khaya senegalensis. Molecules.

[bb0025] Avanza M.V., Álvarez-Rivera G., Cifuentes A., Mendiola J.A., Ibáñez E. (2021). Phytochemical and functional characterization of phenolic compounds from cowpea (Vigna unguiculata (L.) Walp.) obtained by green extraction technologies. Agronomy.

[bb0030] Aviña-Zubieta J.A., Daftarian N., Esdaile J.M. (2025). Dubois’ Lupus Erythematosus and Related Syndromes.

[bb0035] Ayo D., Odongo B., Omara J., Andolina C., Mulder O., Staedke S.G., Bousema T. (2021). Plasmodium malariae infections as a cause of febrile disease in an area of high plasmodium falciparum transmission intensity in eastern Uganda. Malar. J..

[bb0040] Badock E.A., Niang L., Dramé A., Nkounkou-Loumpangou C., Touré O., Sokhna O., Ayessou N.C., Cissé M. (2024). Phytochemical screening and assays of phenolic compounds in Senna occidentalis L. leaf and seed extracts. Food Nutr. Sci..

[bb0045] Bayang J.P., Touwang C., Mamoudou H., Woudam E.S., Koubala B.B. (2025). Variation of nutrients and bioactive compounds of five wild edible leafy vegetables from far north region of Cameroon. Food Chem. Adv..

[bb0050] Duffy P.E., Gorres J.P., Healy S.A., Fried M. (2024). Malaria vaccines: a new era of prevention and control. Nat. Rev. Microbiol..

[bb0055] Espinoza J.L. (2025). Candidate drug repurposing for malaria: perspectives for optimising clinical trials. Lancet Reg. Heal. - Am..

[bb0060] Finney D. (1952).

[bb0065] Hamadou M., Daoudou B., Paul B.M., Mohamadou S., Roger D.D. (2020). Inhibitory effect of methanolic and methanolic-aqueous mixture extract of leaves of Plectranthus neochilus Schltr (Lamiaceae) and Bauhinia rufescens lam (Fabaceae) on two strains of enterobacteria producing Beta-lactamases. J. Adv. Microbiol..

[bb0070] Hamadou M., Martin Alain M.M., Obadias F.V., Hashmi M.Z., Başaran B., Jean Paul B., Samuel René M. (2022). Consumption of underutilised grain legumes and the prevention of type II diabetes and cardiometabolic diseases: evidence from field investigation and physicochemical analyses. Environ. Challenges.

[bb0075] Hoekstra J.A. (1987). Acute bioassays with control mortality. Water Air Soil Pollut..

[bb0080] Kacholi D.S. (2024). A comprehensive review of antimalarial medicinal plants used by Tanzanians. Pharm. Biol..

[bb0085] Kolawole A.O., Okonji R.E., Ajele J.O. (2011). Tithonia diversifolia, Cyperus rotundus and Hyptis suaveloensis ethanol extracts combinatorially and competitively inhibit affinity purified cowpea storage bruchid (Callosobrochus maculatus) glutathione S-transferase. Arthropod Plant Interact..

[bb0090] Leutcha P.B., Mamoudou H., Nganso Ditchou Y.O., Ansari S.A., Amang à Ngnoung G.A., Mujwar S., Domga Taiga J., Agrawal M., Messah Nembot G., Boubakari Hamadou S., Meli Lannang A., Siwe Noundou X. (2025). Flavonoids and other constituents from Jacaranda mimosifolia: in vitro analysis, molecular docking, and molecular dynamic simulations of antioxidant and anti-inflammatory activities. Biomed. Pharmacother..

[bb0095] Liu W., Li Y., Shaw K.S., Learn G.H., Plenderleith L.J., Malenke J.A., Sundararaman S.A., Ramirez M.A., Crystal P.A., Smith A.G., Bibollet-Ruche F., Ayouba A., Locatelli S., Esteban A., Mouacha F., Guichet E., Butel C., Ahuka-Mundeke S., Inogwabini B.-I., Ndjango J.-B.N., Speede S., Sanz C.M., Morgan D.B., Gonder M.K., Kranzusch P.J., Walsh P.D., Georgiev A.V., Muller M.N., Piel A.K., Stewart F.A., Wilson M.L., Pusey A.E., Cui L., Wang Z., Färnert A., Sutherland C.J., Nolder D., Hart J.A., Hart T.B., Bertolani P., Gillis A., LeBreton M., Tafon B., Kiyang J., Djoko C.F., Schneider B.S., Wolfe N.D., Mpoudi-Ngole E., Delaporte E., Carter R., Culleton R.L., Shaw G.M., Rayner J.C., Peeters M., Hahn B.H., Sharp P.M. (2014). African origin of the malaria parasite plasmodium vivax. Nat. Commun..

[bb0100] Loy D.E., Liu W., Li Y., Learn G.H., Plenderleith L.J., Sundararaman S.A., Sharp P.M., Hahn B.H. (2017). Out of Africa: origins and evolution of the human malaria parasites plasmodium falciparum and plasmodium vivax. Int. J. Parasitol..

[bb0105] Lum Nde A., Chukwuma C.I., Erukainure O.L., Chukwuma M.S., Matsabisa M.G. (2022). Ethnobotanical, phytochemical, toxicology and anti-diabetic potential of Senna occidentalis (L.) link; a review. J. Ethnopharmacol..

[bb0110] Mamoudou H., Mune Mune M.A. (2024). Investigating Bambara bean (Vigna subterranea (Verdc.) L.) protein and hydrolysates: a comprehensive analysis of biological and biochemical properties. Appl. Food Res..

[bb0115] Mamoudou H., Başaran B., Mune M.A.M., Abubakar A.M., Nandwa J.O., Raimi M.K.Z., Hashmi M.Z. (2024). Bioactive peptides derived from the enzymatic hydrolysis of cowhide collagen for the potential treatment of atherosclerosis: a computational approach. Intell. Pharm..

[bb0120] Mamoudou H., Obadias F.V., Samuel René M., Martin Alain M.M. (2024). Physical characteristics, chemical composition, and antioxidant properties of defatted grain legumes cultivated in Diamare division (far north region, Cameroon). Appl. Food Res..

[bb0125] Mamoudou H., Abdoulaye A.H., Ditchou N.Y.O., Olumasai J.N., Adissa R.M.Z.K., Mune M.A.M. (2025). Computational investigation of Plectranthus neochilus essential oil phytochemicals interaction with dipeptidyl peptidase 4: a potential avenue for antidiabetic drug discovery. Curr. Pharm. Anal..

[bb0130] Mogaka S., Molu H., Kagasi E., Ogila K., Waihenya R., Onditi F., Ozwara H. (2023). Senna occidentalis (L.) link root extract inhibits plasmodium growth in vitro and in mice. BMC Complement. Med. Ther..

[bb0135] Mogaka S., Mulei I., Njoki P., Ogila K., Waihenya R., Onditi F., Ozwara H. (2023). Antimalarial efficacy and safety of *Senna occidentalis* (L.) link root extract in plasmodium berghei -Infected BALB/c Mice. Biomed. Res. Int..

[bb0140] Muturi E.J., Ramirez J.L., Doll K.M., Bowman M.J. (2017). Combined toxicity of three essential oils against Aedes aegypti (Diptera: Culicidae) larvae. J. Med. Entomol..

[bb0145] Nandwa J.O., Mehmood A., Mahjabeen I., Raheem K.Y., Hamadou M., Raimi M.Z.K.A., Kayani M.A. (2024). miR-4716–3p and the target AKT2 gene/rs2304186 SNP are associated with blood cancer pathogenesis in Pakistani population. Non-Coding RNA Res..

[bb0150] Nganso Ditchou Y.O., Soh D., Nkwengoua Tchouboun E.Z., Tchana Satchet E.M., Mamoudou H., Nyassé B. (2020). Qualitative analysis of peptides and biological activities of Allexis cauliflora (Violaceae) leaves. J. Nat. Prod. Resour..

[bb0155] Nganso Ditchou Y.O., Leutcha P.B., Miaffo D., Mamoudou H., Ali M.S., Amang à Ngnoung G.A., Soh D., Agrawal M., Darbawa R., Zondegoumba Nkwengoua Tchouboun E., Meli Lannang A., Siwe Noundou X. (2024). In vitro and in silico assessment of antidiabetic and antioxidant potencies of secondary metabolites from Gymnema sylvestre. Biomed. Pharmacother..

[bb0160] Nguiffo-Nguete D., Nongley Nkemngo F., Ndo C., Agbor J.-P., Boussougou-Sambe S.T., Salako Djogbénou L., Ntoumi F., Adegnika A.A., Borrmann S., Wondji C.S. (2023). Plasmodium malariae contributes to high levels of malaria transmission in a forest–savannah transition area in Cameroon. Parasit. Vectors.

[bb0165] Oscar Ditchou Nganso Y., Marthe Satchet Tchana E., Doutsing Kahouo A., Gabrielle à Ngnoung Amang A., Abah K., Fomena H., Mamoudou H. (2020). Inhibitory effect and antimicrobial activity of secondary metabolites of *Khaya Senegalensis* (Desr.) A. Juss. (Meliaceae). Sci. J. Chem..

[bb0170] Oscar Ditchou Nganso Y., Sidjui Sidjui L., Gabrielle A Ngnoung Amang A., Doutsing Kahouo A., Abah K., Fomena H., Hamadou M. (2020). Identification of peptides in the leaves of Bauhinia rufescens Lam (Fabaceae) and evaluation of their antimicrobial activities against pathogens for aquaculture. Sci. J. Chem..

[bb0175] Peard E.F., Luu C., Hageman K.J., Sepesy R., Bernhardt S.A. (2024). Exploring sources of inaccuracy and irreproducibility in the CDC bottle bioassay through direct insecticide quantification. Parasit. Vectors.

[bb0180] Raimi M.Z.K.A., Nadeem A., Raheem K., Hussain G., Zafeer N., Hamadou M., Irfan M., Nandwa J., Ahmad F., Ullah A., Shabbir A. (2024). In silico analysis of RPS4X (X-linked ribosomal protein) with active components from black seed (Nigella sativa) for potential treatment of multiple sclerosis. J. Mol. Struct..

[bb0185] Sarma R., Adhikari K., Mahanta S., Khanikor B. (2019). Combinations of plant essential oil based terpene compounds as larvicidal and adulticidal agent against Aedes aegypti (Diptera: Culicidae). Sci. Rep..

[bb0190] Snow R.W., Sartorius B., Kyalo D., Maina J., Amratia P., Mundia C.W., Bejon P., Noor A.M. (2017). The prevalence of plasmodium falciparum in sub-Saharan Africa since 1900. Nature.

[bb0195] Tang Jiandong, Li R., Wu B., Tang Junrong, Kan H., Zhao P., Zhang Y., Wang W., Liu Y. (2024). Secondary metabolites with antioxidant and antimicrobial activities from Camellia fascicularis. Curr. Issues Mol. Biol..

[bb0200] Wangrawa D.W., Yaméogo F., Sombié A., Esalimba E., Ochomo E., Borovsky D., Badolo A., Sanon A. (2023). Methanol and acetone extracts from the leaves of selected aromatic plants affect survival of field collected Anopheles arabiensis (Diptera: Culicidae) from Kisumu, Kenya. J. Med. Entomol..

[bb0205] Wangso H., Laya A., Leutcha P.B., Koubala B.B., Laurent S., Henoumont C., Talla E. (2022). Antibacterial and antioxidant activities and phytochemical composition of Stereospermum kunthianum root bark. Nat. Prod. Res..

[bb0210] WHO (2005).

[bb0215] WHO (2013).

[bb0220] WHO (2015).

[bb0225] WHO (2016).

[bb0230] Youssefi M.R., Tabari M.A., Esfandiari A., Kazemi S., Moghadamnia A.A., Sut S., Dall’Acqua S., Benelli G., Maggi F. (2019). Efficacy of two monoterpenoids, carvacrol and thymol, and their combinations against eggs and larvae of the West Nile vector Culex pipiens. Molecules.

